# Mechanisms of picosecond laser-induced damage in common multilayer dielectric coatings

**DOI:** 10.1038/s41598-018-37223-0

**Published:** 2019-01-24

**Authors:** Alexei A. Kozlov, John C. Lambropoulos, James B. Oliver, Brittany N. Hoffman, Stavros G. Demos

**Affiliations:** 1 Laboratory for Laser Energetics, University of Rochester, 250 East River Road, Rochester, NY 14623-1299 USA; 2 Department of Mechanical Engineering, University of Rochester, 235 Hopeman Building, Rochester, NY 14627 USA

## Abstract

The physical mechanisms and ensuing material modification associated with laser-induced damage in multilayer dielectric high reflectors is investigated for pulses between 0.6 and 100 ps. We explore low-loss multilayer dielectric SiO_2_/HfO_2_ mirrors which are commonly employed in petawatt-class laser systems. The spatial features of damage sites are precisely characterized, enabling the direct correlation of the observed damage morphology to the location of energy deposition and the corresponding standing-wave electric-field intensities within the layer structure. The results suggest that there are three discrete damage-initiation morphologies arising from distinctly different mechanisms: the first prevailing at laser pulse lengths shorter than about 2.3 ps, while the other two are observed for longer pulses. Modeling of the thermomechanical response of the material to localized laser-energy deposition was performed for each type of damage morphology to better understand the underlying mechanisms of energy deposition and subsequent material response.

## Introduction

The capability of multilayer dielectric (MLD) mirrors to transport ultrahigh-intensity laser pulses is limited by laser-induced damage that is associated with the localized formation of a plasma accompanied by high temperatures and pressures. The morphology of the damage sites captures thermomechanical signatures of the material relaxation pathway, which in turn can be used to extract information about the damage-initiation mechanism. We use an array of imaging modalities to capture the spatial dimensions and characteristic attributes of damage sites formed under excitation between 0.6 and 100 ps. We also investigate the mechanisms of laser-induced damage on low-loss MLD SiO_2_/HfO_2_ mirrors with well-understood standing-wave electric-field intensities within the layer structure. We have observed and modeled three general damage morphologies that are largely associated with the geometry of plasma-confinement conditions and the duration of the laser pulse. For pulses shorter than about 2.3 ps, damage is initiated by electric-field–induced volume breakdown. For pulses between 2.3 and 100 ps, damage arises from isolated nanoscale defect structures, and material failure is governed by high-pressure or temperature effects, depending on the depth of these defects.

Laser technology has rapidly evolved during the past three decades, and the spectrum of related applications has been continually expanding. Powerful laser systems have been developed for use in basic research (such as to study extreme states of matter or in an effort to create fusion in a laboratory setting) as well as civilian and military applications^1,2^. The limiting factor governing the output power of such laser systems is typically related to the ability of the constituent optical components to handle the generated optical energy^3^.

Laser damage is associated with a defect-assisted laser-induced breakdown process^4^ that results in the rapid formation of near-solid-state–density plasma and transition of the affected material to a warm-dense-matter (WDM) state. Laser-induced damage in optical materials differs from laser-induced ablation of materials used in various applications (such as micromachining) by the fact that it is defect driven and occurs at laser fluences below the classical ablation threshold of the material. As a result, damage sites are typically created on the surface of the optic (due to higher exposure to contaminants during the manufacturing process) and are smaller than the size of the laser beam impinging on the optic, with their size related to the size of the damage-initiating defect and the duration of the laser pulse. Temperatures and pressures of the order of 1 eV and 10 GPa, respectively, can be generated during laser-induced damage under nanosecond irradiation^5^, while the peak pressure for a given pulse energy increases with decreasing pulse length and can reach values of the order of 100 GPa or larger^6^. The morphology of the damage sites provides evidence of the subsequent material relaxation. This enables one to accurately determine the location of energy deposition and, with lesser accuracy, the thermodynamic pathway of material relaxation. The latter is embodied by thermomechanical signatures of the damage site such as the volume of material removed, the depth of the damage site, the presence of remaining melted material, and the extent of the mechanical damage to the surrounding material. In general, damage initiates from defect-based absorption leading to isolated damage sites of the order of a few *μ*m for pulse durations longer than about 100 ps, while electric-field–induced volume breakdown (such as defect-assisted multiphoton absorption) initiates damage for pulses shorter than about 1 ps^7–11^. The mechanism involved for intermediate pulse durations (between about 1 and 100 ps) remains inadequately understood, especially for complex optical structures such as MLD’s. Such structures involve more than one material (typically alternating high- and low-refractive-index layers within the MLD stack), while damage can initiate within the different layers. It has been previously reported that three general morphologies of damage sites form in a vacuum environment in SiO_2_/HfO_2_ MLD high reflectors (as depicted in Figs 6–8 by Kozlov *et al*. in ref.^12^). The first two morphologies were previously reported, and it was suggested that they involve different damage-initiation mechanisms^11,13^. More-recent work has confirmed these observations^14,15^.

The aim of this work is to provide a detailed description of the damage morphology in common MLD high reflectors for pulses between 0.6 and 100 ps (full width at half maximum) and identify the underlying damage-initiation mechanism. We explore low-loss multilayer dielectric SiO_2_/HfO_2_ mirrors that are typically employed in petawatt-class laser systems operating at high peak intensity and energy per pulse in order to study laser–matter interactions in extreme conditions.

The detailed results obtained in this work suggest that while the first damage morphology (labeled as type I) occurs at laser pulse lengths shorter than about 2.3 ps, the other two morphologies (labeled as type II and III) are observed for longer pulses and can be simultaneously manifested within the same laser irradiated area. Modeling of the thermomechanical response of the material to localized laser-energy deposition for each type of damage morphology helps reveal the underlying mechanism of material modification and failure. This improved understanding, in turn, can be used to help design and fabricate materials with higher damage thresholds. This effort is motivated by the need to improve the performance and reduce the operational cost of laser systems such as OMEGA EP located at the University of Rochester’s Laboratory for Laser Energetics, operating at a wavelength of 1053 nm with an adjustable pulse duration between 0.7 and 100 ps.

## Methods

The damage-test laser system used in this study has been described in detail elsewhere^12,16^. The laser is operating at 1053 nm, and the pulse duration is adjustable between 600 fs and 100 ps. The laser beam is focused on the sample using a 200-cm-focal-length mirror resulting in a nearly circular ~350-*μ*m-diam beam spot measured using the 10/90 knife-edge method. The laser is operated in single-shot mode with each tested location on the sample exposed to a single pulse at a predetermined fluence. Using a beam splitter, a small part of the laser beam was directed to an energy meter and laser beam profiler that was set up to capture the beam at an equivalent optical plane to the location of the sample. This system was calibrated before each experiment and provided the spatial distribution of the laser energy on the sample for every shot. The laser fluence is reported as the peak fluence at the center of the irradiated area. During the experiments, the laser fluence at each pulse length was gradually increased until a material modification (which was classified as laser induced damage) was observed *in situ* using an on-line microscopic imaging system. The damage detection system has about 2 *μ*m of optical resolution and is based on subtraction of the sample images acquired before and after laser exposure. This enables detection of the sample modifications with spatial dimensions smaller than the optical resolution of the imaging system.

The experiments were performed in a vacuum chamber at a pressure of ~10^–6^ Torr. The results reported in this work were obtained from samples representing three SiO_2_/HfO_2_ MLD mirror designs fabricated in our own coating facilities via electron-beam evaporation. The first design is a 28-layer quarter-wave stack for *s*- and *p*-polarized reflection at 29° incidence. The second and third designs consist of 20 layers for *s*-polarized reflection at 55.4° and 45°, respectively. The top layer in all designs is a half-wave optical thickness of silica.

The morphologies of laser-induced damage sites generated at various pulse lengths were characterized using an array of imaging modalities including AFM and SEM, with particular emphasis on measuring the depth of the features generated around the onset of damage. The depth measurement was used as a diagnostic to identify the location of the initial energy deposition within the MLD stack and enable correlation with the corresponding electric-field distribution.

The measurements indicate that type-I damage sites initiate in the first SiO_2_ layer for *s*-polarized pulses and at the second SiO_2_/HfO_2_ interface (first HfO_2_ and second SiO_2_ layer) for *p*-polarized pulses for all MLD designs used in this work. On the other hand, type-II damage sites initiated at the first SiO_2_/HfO_2_ interface for both polarizations for all MLD designs tested. This is the location within the first HfO_2_ layer that is exposed to the maximum electric-field intensity. Finally, all type-III damage sites are confined within the top 150 nm of the first SiO_2_ layer. The laser fluence required to initiate damage on each sample varies depending on MLD design, laser polarization, and angle of incidence. In general, the damage-threshold fluence increases with pulse length. This issue is discussed in more detail later.

**Figure 1:**
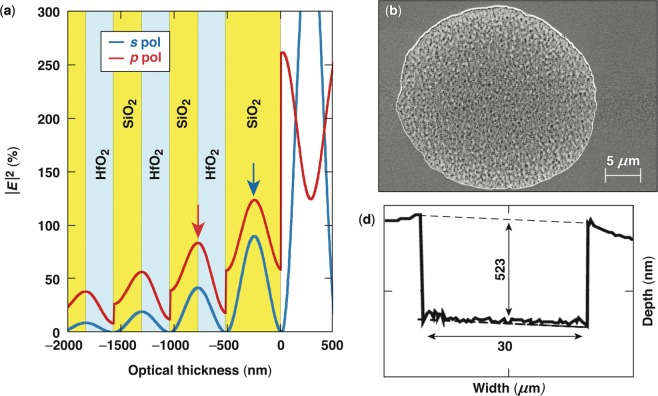
(**a**) Depiction of the electric-field profile within the outer layers of the coating from design 2 as a function of the optical thickness. The other designs used in this work have similar profiles. (**b**) Scanning electron microscopy (SEM) image of a typical type-I damage site formed under *p*-polarized pulses. (**c**) Lineout along the center of a damage site obtained from its atomic force microscopy (AFM) image.

The electric-field intensity distribution within the MLD stack was calculated using commercially available software (Optilayer). The electric-field profiles within the multilayer stack for all three designs used in this work are very similar. As an example, Fig. 1a depicts the electric-field profile within the top six layers for design 2 as a function of the optical thickness, starting from the air interface of the MLD coating. The positions of the alternating SiO_2_ and HfO_2_ layers are also shown. Within these designs, the first peak of the electric field for both polarizations is within the top SiO_2_ layer, and the second peak is at the interface between the first HfO_2_ layer and the second SiO_2_ layer. The local field enhancement has implications on the laser-damage behavior of the materials and is discussed within this work.

## Experimental Results

Figure 1b shows a top-view SEM image of a type-I damage site demonstrating well-defined edges and a circular profile that is centered at the location of peak intensity within the laser beam. This morphology is observed for the shortest pulse durations, of the order of ~2.3 ps or less. It is characterized by a large shallow pit having a diameter that corresponds to the area of peak intensity of the laser spot and specifically to the area of about 95% of the peak intensity, which has a radius of approximately 15 *μ*m in the experimental system used for this work. Depending on the polarization of incident light, the base of the pit (bottom of crater) was located either within the top SiO_2_ layer or at the interface between the first HfO_2_ and the second SiO_2_ layer (locations are indicated with arrows in Fig. 1a). These positions correlate very precisely (within less than 10 nm) to the depth predicted by the model for damage initiation by Hervy *et al*.^17^. Higher-magnification SEM and AFM images reveal that the sidewalls are nearly vertical while the bottom of the pit (crater) is coarse with signs of melted material formed during laser damage. The crater morphology is discussed in more detail later. Figure 1c shows the lineout obtained from an AFM image of a damage site formed under *p*-polarized pulses capturing the depth of the pit, the crater roughness, and the geometry of the sidewall. It should be noted that, in order to correlate the measured (geometric) depth of the pit to the optical thickness predicted by the standing-wave electric-field profile in Fig. 1a, the depth must be scaled by the refractive index of the material(s) removed.

The morphology of a type-I damage site suggests that plasma forms at the location of peak intensity of the laser beam covering (within our experimental system) a circular region with an ~15-*μ*m radius. This represents a narrow range of electric-field intensities (within the laser beam profile irradiating the sample) that can support plasma formation (e.g., via multiphoton absorption). Using the same range of electric-field intensities as calculated along the *z* axis (inside the coating) and assuming that the same mechanism applies for the ionization of the material, the thickness of the ionized volume (plasma at the onset of damage initiation) is estimated to be 60 to 80 nm. This approach is similar to that adopted by Kumar *et al*.^18^. The morphology of the damage site implies that the generated pressure is sufficient to support shear fracture and detachment of the overlying layer. Subsequent rapid cooling results in remnants of liquid material.

**Figure 2:**
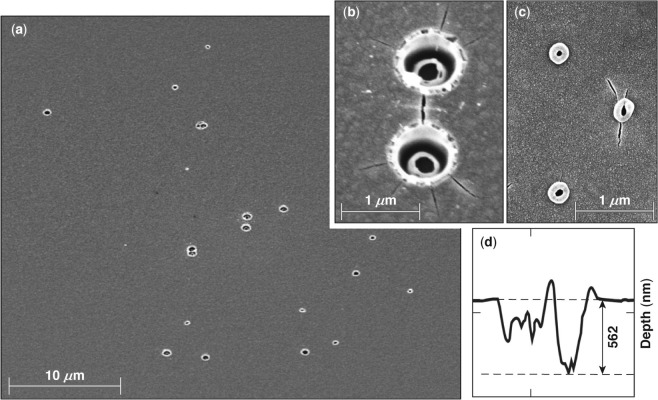
SEM images of type-II damage sites formed under irradiation with (**a**,**b**) 20-ps pulses and (**c**) a 4.5-ps pulse. (**d**) An AFM line scan through two adjoined damage sites revealed the depth of the crater, which is at the interface between the second and third layers.

Figure 2 captures the typical morphology of the second type of damage site (type II) observed under excitation with pulses longer than ~2.3 ps. Figure 2a shows that the damage sites are isolated (and not correlated to “hot spots” on the laser beam), indicating that they originate from nanoscale defects. The size of these sites is of the order of 1 *μ*m in diameter, depending on pulse length. A higher-magnification SEM image of two neighboring damage sites, formed under irradiation with a single 20-ps pulse (shown in Fig. 2b), reveals a complex crater morphology containing a venting hole and one or more inner quasi-spherical shells. This complex structure, accompanied by the presence of a significant amount of melted material, indicates a gradual cooling process after the energy is deposited. In addition, the craters are surrounded by radial cracks of the order of 1 *μ*m in length, indicating the presence of tensile hoop stresses surrounding the crater region. Figure 2c shows an SEM image of a damage site formed under 4.5-ps excitation. The features observed are similar, including the presence of a vent opening and radial cracks, but the spatial dimensions are smaller. In general, the size of type-II damage sites is found to be proportional to the pulse length of the laser illumination. (Additional discussion is provided in the next section.)

AFM measurements on isolated type-II damage sites could not resolve the bottom of this complex structure. However, occasionally two or more sites were in sufficiently close proximity such that a larger horizontal extent of material was removed. Such sites were used to measure the damage depth using AFM, indicating the damage crater extended to the interface between the first HfO_2_ and second SiO_2_ layer for all samples used in this work.

The depth of type-II damage sites suggests that the defect structures initiating damage are located within the first HfO_2_ layer at the position of peak electric-field intensity, which is near the interface with the second silica layer. These defect structures absorb a sufficient amount of energy to form plasma, but the pressure generated is insufficient to rupture the layer above, as in type-I damage sites. As a result, the absorbed energy is dissipated via heat diffusion, which leads to a softening of the top layer. In addition, the generated gaseous material expands, producing swelling on the surface above the location of energy deposition, generating tensile hoop stresses and cracking the still-cold top layer. The combination of softening and cracking leads to the formation of a venting path where the gaseous and liquid material is released.

The complex morphology of the damage sites can be attributed to the very different thermodynamic properties of SiO_2_ and HfO_2_ layers. Specifically, the melting temperature of the hafnia is very close to the evaporation temperature of the silica. Consequently, melting of a hafnia layer should be accompanied by evaporation of material from the adjacent silica layer. Therefore, we postulate that the inner shell observed in the damage sites is the hafnia layer involved in the damage process with a venting path for release of the evaporated material of the underlying silica layer.

**Figure 3:**
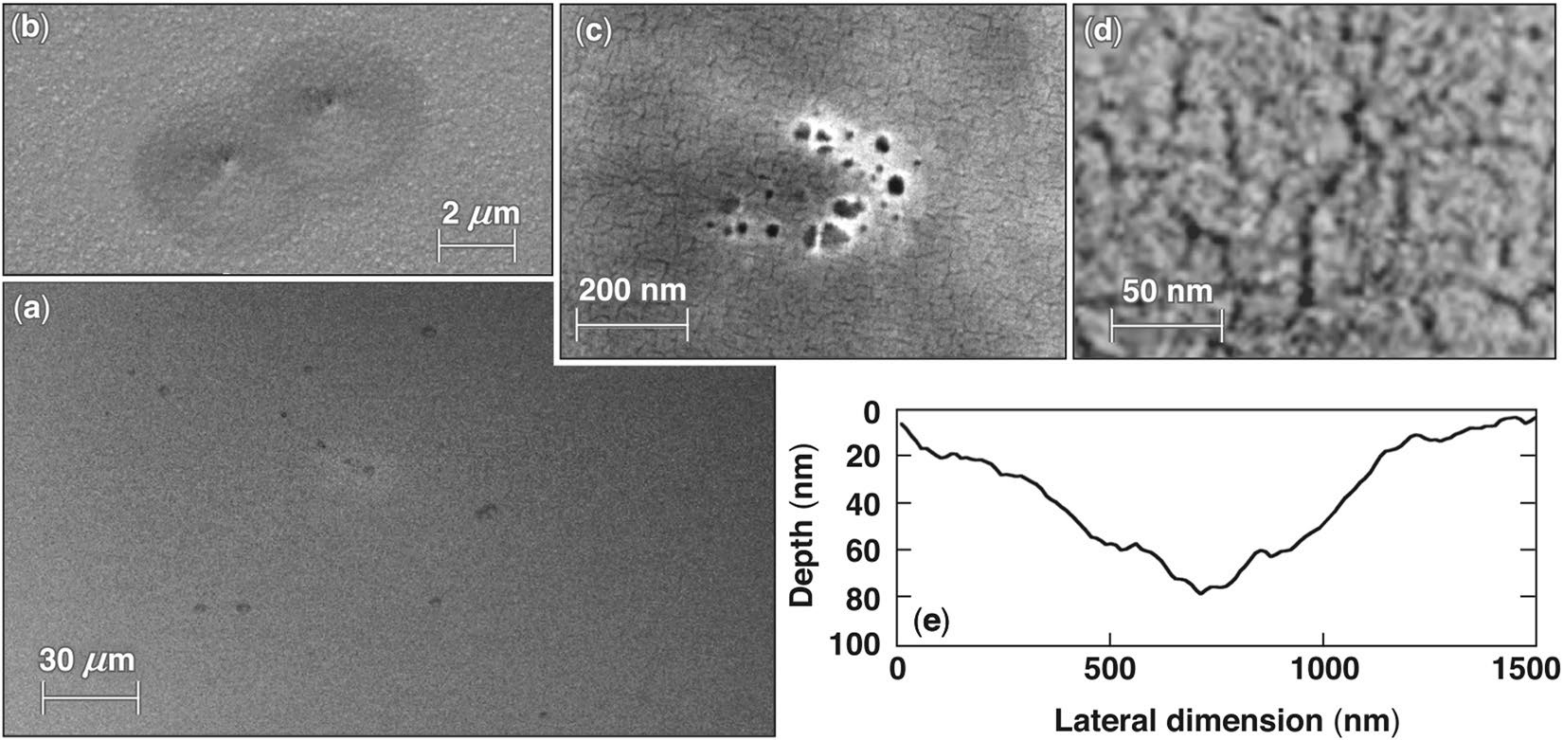
Images of typical type-III damage sites. (**a**–**d**) SEM images of type-III damage sites at different magnifications formed under irradiation with (**a**) a 50-ps pulse and (**b**–**d**) 20-ps pulses. Images (**c**) and (**d**) are from the center of the damage crater for fluences 10% and 1% above the nominal laser-induced damage threshold (LIDT), respectively. (**e**) The AFM line scan through a damage site captures the high aspect ratio of this morphology.

The third damage morphology (type III) was also observed for pulse lengths longer than ~2.5 ps. The damage consists of isolated sites within the illuminated region, each of which is a shallow quasi-conical crater having a diameter of ~2 to 3 *μ*m. Figure 3a shows a lower-resolution SEM image of a region that contains a distribution of multiple damage sites formed under excitation with 50-ps pulses, suggesting damage initiation arises from isolated defect structures. Figure 3b shows a higher-magnification SEM image capturing two adjoining damage sites generated with 20-ps pulses. Their visualization in SEM images is rather difficult because of the smooth surfaces of the craters. However, a darker feature in the middle (bottom) of the crater is better visualized. A high-magnification SEM image of the bottom of two different craters generated with 20-ps pulses having nominal fluences of about 10% and 1% above the damage threshold fluence are shown in Fig. 3c,d, respectively. The presence of a network of cracks having a width of the order of a few nanometers is visible at the bottom of type-III craters along with what appear to be quasi-spherical voids having diameters of the order of 10 nm that are visible only in sites that were formed using a laser fluence exceeding the LIDT. We postulate that these features are related to material modification (following plasma formation) in the region surrounding the damage-initiating defect. The depth of the craters was best captured using AFM imaging. Figure 3e shows a cross section along the center of a type-III damage site imaged using AFM. In general, the depth of this type of damage site is of the order of 150 nm or less. A characteristic trait of type-III damage sites is that their depth is not correlated with the electric-field–intensity peak.

The formation of type-III damage sites, consisting of quasi-conical craters and a central-region morphology suggestive of an explosive boiling process, may be associated with pressure-driven material ejection; this is similar to that involved in the formation of type-I damage sites. The volume of the plasma region in this case depends on the size of the sub-micrometer defect structures; consequently, the total generated pressure energy is much smaller compared to that generated during formation of type-I damage sites. Since the energy required to create such high aspect ratio conical craters is strongly dependent on the depth of the crater (the area of new surface formed), only defects that are relatively large and located closer to the surface may generate a sufficient amount of energy to support formation of type-III damage sites. This is consistent with the observed limit in depth (<150 nm) for type-III damage sites. We therefore anticipate that such defects, when located deeper in the MLD structure, may create small voids containing melted and densified material.

### Modeling

The underlying mechanisms associated with the experimentally observed damage morphologies are investigated using micro- and nanomechanical models of the material’s response to the generation of high pressure and temperature during damage at different pulse durations. Toward this goal, we need to assess the thermomechanical properties of the silica and hafnia layers comprising the MLD, as well as the relevant thermomechanical time scales in relation to the laser pulse width. An estimate of the elastodynamic time scale can be obtained by using the stress-wave speed in silica of 5700 m/s, leading to times of 2 to 10 ps to travel distances of 10 to 50 nm. On the other hand, thermodynamic time scales can be estimated by using a thermal diffusivity of 0.7 × 10^−6^ m^2^/s, leading to thermal time scales of 40 to 900 ps for thermal diffusion over distances of 10 to 50 nm.

It is well known that the mechanical properties of nanometer-scale layers are affected by the deposition process, the microstructure of each layer, and the presence of multiple interfaces^19^. For a silica layer, we assume a uniaxial yield stress of 4 GPa based on the nano-indentation work by Mehrotra *et al*.^20^. For elastic properties, we assume bulk properties for Young’s modulus (72 GPa for silica and 300 GPa for hafnia) and Poisson’s ratio (0.17 for silica and 0.25 for hafnia). For the thermal properties, we assume for our simulations the bulk values: thermal conductivities of 1 and 2 W/m.K, mass densities of 2200 and 9500 kg/m^3^, and heat capacities of 750 and 270 J/kg.K for silica and hafnia, respectively. We emphasize that, especially for the thermal conductivity, a well-documented dependence on film thickness is reported in the literature^21,22^. For silica thin films, thermal conductivities range from 0.7 to 1.2 W/m.K, i.e., comparable to the bulk value. The values for hafnia vary from 0.4 to 2.6 W/m.K (ref.^22^). We consider the above values to be sufficiently accurate to plausibly model the mechanisms of damage formation and to interpret the experimental observations.

### Modeling mechanism of type-I damage sites

**Figure 4:**
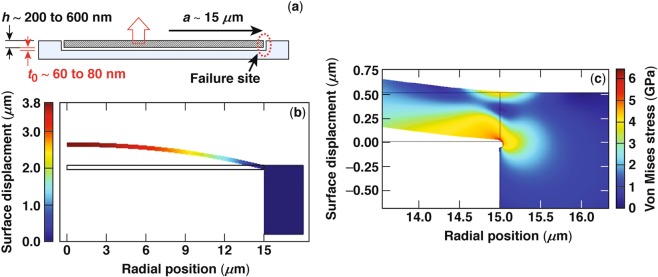
Depiction of the mechanism involved in the formation of type-II damage sites. (**a**) Plasma forms within a thin region of width *t*_0_ and induces mechanical pressure *p*, which inflates the overlying layer with thickness *h*. (**b**) Finite-element calculation of silica blister inflation assuming *h* = 200 nm and pressure *p* = 45 MPa. (**c**) Formation of a plastic hinge near the support point of the membrane.

Based on the experimental results discussed in the previous section, we assume plasma formation within a thin region of thickness *t*_0_ below the surface (as a result of breakdown caused by localized peak-electric-field intensity), followed by the evaporation of material and building of a pressure *p*. This in turn gives rise to the formation of a circular membrane, or blister, of radius *a* and thickness *h* as depicted in Fig. 4a. The pressure *p* induces an inflation of the material above, described by a center deflection of magnitude *w*_c_. The center deflection scales with thickness *h* and material properties as^23^

1 $$({w}_{{\rm{c}}}/h)+A{({w}_{{\rm{c}}}/h)}^{3}=B(p/E){(a/h)}^{4},$$

where *E* is Young’s modulus of the materials. A finite element model was implemented in Comsol (Version 5.3). The finite element axisymmetric boundary conditions consist of a vertical displacement at the center of the blister, while the edge of the blister is attached to the remaining elastic material via a shallow notch of radius 80 nm (used to model the separation of the blister). The model allows fully for nonlinear strains, necessary to capture the large blister deflections. The blister and all supporting material were modeled as elastoplastic, with the properties given above, and allowing for a small tangent modulus so as to expedite convergence. The blister was deformed by applying uniform pressure under the blister. A systematic study was conducted to ensure that there was no dependence on discretization. Figure 4b shows the finite-element calculation result of inflation of an axisymmetric membrane of thickness *h* = 200 nm under pressure *p* = 45 MPa, leading to center deflection *w*_c_ = 3.81 *μ*m. This result is based on elastic behavior of the membrane material. The constants *A* and *B* are entirely dependent on Poisson’s ratio of the material. The calculation also shows the formation of a plastic hinge near the support point of the membrane, as shown in Fig. 4c.

**Figure 5:**
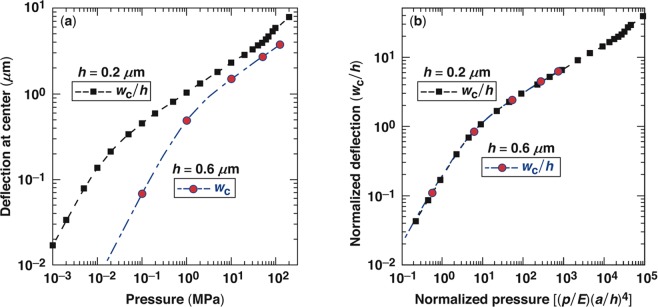
Finite-element predictions of the center deflection of an inflated membrane by pressure *p* for two membrane thicknesses of 200 and 600 nm and radius *a* = 15 *μ*m. (**a**) The center deflection *w*_c_ as a function of the pressure and (**b**) the normalized center deflection *w*_c_/*h* is a function of the dimensionless pressure.

Additional finite-element numerical calculation results are shown in Fig. 5 for two different layer (membrane) thicknesses. Specifically, Fig. 5a shows the center deflection *w*_c_ of the inflated membrane having a radius *a* = 15 *μ*m and Young’s modulus *E* = 70 GPa for two membrane thicknesses of *h* = 200 and *h* = 600 nm. Furthermore, Fig. 5b shows a normalized center deflection (dimensionless) represented as *w*_c_/*h* as a function of a dimensionless pressure represented as (*p/E*)(*a/h*)4. This normalization reveals that the results for different *h* collapse on a single profile as expected based on ref.^23^. Extrapolations to large or small pressures easily give the fitting constants as *A* = 0.60 and *B* = 0.19.

Several important aspects are apparent from this model. For small center deflections, i.e., ${w}_{{\rm{c}}}\ll h,$ *w*_c_ scales linearly with pressure, *w*_c_ ∝ *p* since the material behaves in a linear elastic manner, and the strains are small. For larger deflections, i.e., ${w}_{{\rm{c}}}\gg h,$ the scaling is nonlinear and *w*_c_ ∝ *p*1/3 since the strains (while still elastic) are nonlinear. Furthermore, for small pressures and deflections, the numerical results show that the overall shape of the deflected membrane is well approximated by $w(r)={w}_{{\rm{c}}}{(1-{r}^{2}/{a}^{2})}^{2}$, i.e., the slope vanishes at the support point. On the other hand, for larger pressures, the shape is essentially spherical and given by $w(r)={w}_{{\rm{c}}}(1-{r}^{2}/{a}^{2})$.

**Figure 6:**
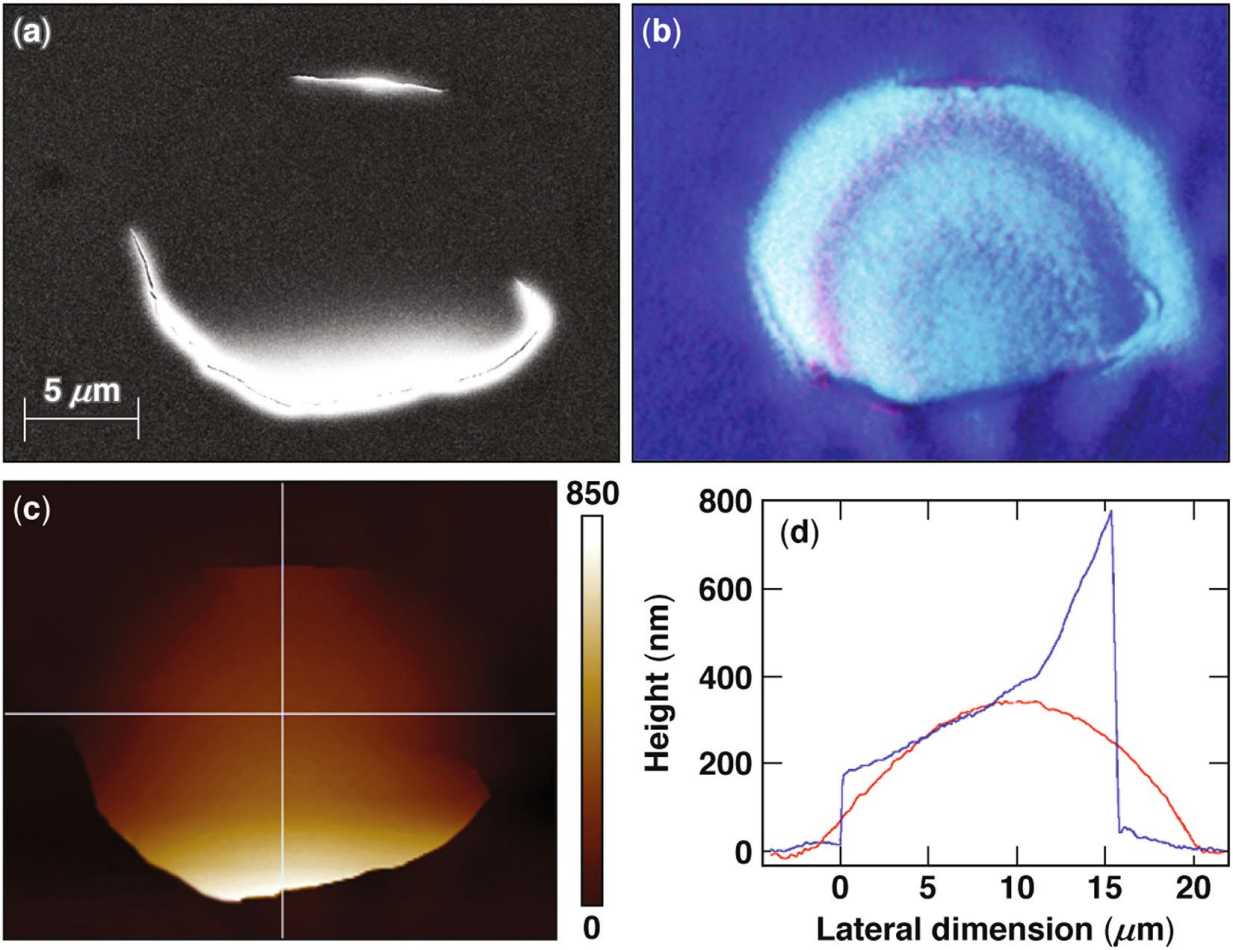
Images of the same damage site generated under 600-fs pulsed irradiation, where the membrane following plasma formation was only partially ruptured (**a**) SEM, (**b**) optical micrograph, (**c**) AFM, and (**d**) the AFM line scan along the horizontal (red line) and vertical (blue line) directions passing through the middle of the damage site.

The numerical simulations demonstrate that the stresses at the support point of the inflated membrane always exceed those at the apex, so that any failure is expected to occur at the support point. To explore the validity of this prediction, we performed additional experiments where the laser fluence was kept at or slightly below the nominal damage threshold. The aim was to generate a plasma-formation event, where the generated pressure was insufficient for the complete removal of the membrane but possibly sufficient to generate an observable material modification. We were able to generate such atypical damage sites under *p*-polarized pulses, with a characteristic example shown in Fig. 6. Specifically, Fig. 6a shows the SEM image of such an “incomplete” damage site, while Fig. 6b shows the corresponding optical micrograph of this site. Both images indicate that the membrane has ruptured at the support point at the top and bottom of the image but not on the sides. Figure 6c shows the same damage site using AFM imaging, indicating that the site ruptured along two asymmetrical arches at the upper and lower edges. The lineouts shown in Fig. 6d along the horizontal and vertical directions (red and blue profiles, respectively) passing through the middle of the damage site confirm that the rupture was partial and that the membrane is still attached at the left and right edges. It is interesting to note that the membrane is lifted at the center by about 350 nm and by about 800 nm at the lower rupture point. In addition, the profile of the damage site along the rupture points does not precisely follow the beam-intensity profile, which may suggest that the rupture occurred at a lower pressure because of structural defects of the coating, thereby facilitating partial failure only. However, the results shown in Fig. 6 demonstrate that the failure mechanism is associated with a rupture at the support point following the formation of a blister, as predicted by the model.

The observed damage site in Fig. 6 can be interpreted via the model predictions in Eq. (1). Using blister size 2*a* = 20 *μ*m and thickness *h* = 0.6 *μ*m with an uplift of 0.35 *μ*m allows one to estimate the blister pressure *p* in the range of 3.5 to 4.5 MPa for the bulk or monolayer Young’s modulus, respectively. An uplift of 0.8 *μ*m would imply a pressure of 13 to 17 MPa.

To further quantify the onset of failure, we observe that at a certain pressure, a plastic hinge occurs at the support point as shown in Fig. 4c. Although the plastic hinge formation arises at a pressure that scales with the layer thickness, our numerical simulations show that the center deflection is between 3 and 4 *μ*m for both layer thicknesses. On the other hand, the pressure required for the hinge formation approximately scales with the layer thickness. This can be easily seen by equilibrating the forces in the vertical direction in Fig. 4a, leading to

2 $$p(\pi {a}^{2})={\tau }_{{\rm{Y}}}(2\pi a)h,$$

where *τ*_Y_ is the yield stress in shear, which is of the order of 2 to 2.3 GPa.

The results in Fig. 5 essentially represent a peak-to-valley (p–v) behavior for the inflated layer. The p–v work can be found by integration. This energy is provided by the plasma formed during damage initiation over the volume (*πa*^2^*t*_0_) of material of thickness *t*_0_. To place in perspective the energy required to form a blister and then a type-I damage site, an equivalent conduction-band electron density is estimated, where the energy from relaxation to the ground state provides the energy for the p–v work involved in blister formation. Table 1 summarizes the model predictions for samples 1 and 2 and for both *s*- and *p*-polarized configurations. The range in pressures corresponds to the range over which the plastic hinge at the support point develops. These large pressures are due to the fact that the plastic hinge at the membrane support periphery penetrates through the entire thickness in the material. The results in Table 1 suggest that relaxation of conduction-band electrons with a density of the order of 10^20^ electrons/cm^3^ is sufficient to provide the energy for type-I damage sites. As a point of reference, the thermal energy required to heat quartz from room temperature to the melting point corresponds to a density of 2.3 × 10^21^ electrons/cm^3^.

**Table 1:** Model predictions at failure of the membrane for the pressure, center deflection, and corresponding electron density required to match the “peak-to-valley” work.

Sample	Pressure (MPa)	Deflection (*μ*m)	Electron density (cm^−3^)
Sample 1, *s*-pol	23 to 55	3 to 4	1 × 10^20^ to 3.2 × 10^20^
Sample 1, *p*-pol	70 to 150	3 to 4	2.3 × 10^20^ to 6.9 × 10^20^
Sample 2, *s*-pol	25 to 60	3 to 4	0.9 × 10^20^ to 2.7 × 10^20^
Sample 2, *p*-pol	75 to 150	3 to 4	3.5 × 10^20^ to 10.4 × 10^20^

### Modeling mechanism of type-II damage sites

**Figure 7:**
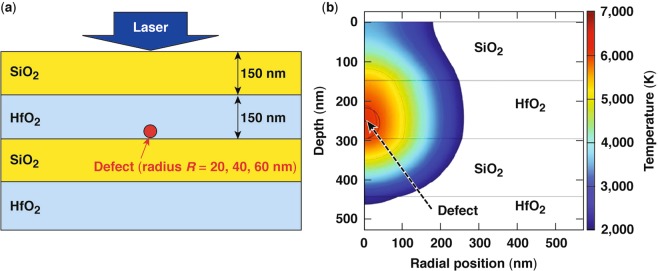
(**a**) Depiction of an absorbing defect and multilayer dielectric (MLD) layers as considered in the modeling of type-II damage sites. (**b**) Temperature distribution at 18-ns delay assumes a SiO_2_/HfO_2_ MLD with the defect located at the second interface having a radius of 40 nm for an incident pulse width of 50 ps and fluence of 15 J/cm^2^.

SEM images of type-II damage sites indicate the presence of modifications resulting from high temperature and pressure. We therefore explore a thermal model of an absorbing defect located near the bottom of the first hafnia layer, as depicted in Fig. 7a (refs^4,5,24–27^). The interaction of the defect with the incident laser radiation leads to plasma formation during the laser pulse which results in a very high localized absorption. In addition, the size of the plasma “ball” expands during the laser pulse^28,29^. Because both the value of the absorption coefficient and the size of the absorber change during the laser pulse, we will assume for simplicity that the defect absorbs all laser light. This overestimation of the energy coupling efficiency may be associated with an underestimation of the size of the absorber. Following plasma formation and termination of the laser pulse, energy relaxation involves thermal diffusion governed by the thermal diffusivity of hafnia and silica. We assume that the defect absorbs thermal power according to its cross-sectional area $\pi {R}_{{\rm{defect}}}^{2}$ and distributes the thermal power over its volume $(4/3)\pi {R}_{{\rm{defect}}}^{{\rm{3}}},$ so that the power absorbed per unit volume has a Gaussian temporal dependence:

3a $$g(t)={g}_{\max }\exp {[-2(t-{t}_{{\rm{peak}}})/{t}_{{\rm{width}}}]}^{2}$$

with

3b $${g}_{\max }=(3/2\sqrt{\pi })F/({t}_{{\rm{width}}}{R}_{{\rm{defect}}}).$$

In this expression, *F* is the laser fluence [J/cm^2^], *t*_peak_ is the time when the pulse is at its peak intensity, and *t*_width_ is the pulse width. Our simulations used *R*_defect_ = 20, 40, and 60 nm and pulse widths of 10 to 50 ps. We have used the bulk properties for SiO_2_ (mass density *ρ* = 2200 kg/m^3^, heat capacity *Cp* = 750 J/kg.K, and thermal conductivity *k* = 1 W/m.K) and HfO_2_ (*ρ* = 9500 kg/m^3^, *Cp* = 280 J/kg.K, and *k* = 2 W/m.K).

The numerical modeling result in Fig. 7b shows the temperature distribution at an 18-ns delay resulting from localized thermal absorption by the defect, assuming the incident pulse width is 50 ps, *R*_defect_ = 40 nm, and the laser fluence is equal to the damage threshold (based on experimental observations) of 15 J/cm^2^. The thermal model used corresponds to classical heat conduction obeying the Fourier law of heat conduction. The isotherms show that the surface temperature reaches a maximum of about 3500 K, which is well above the melting point of silica. The presence of the free MLD surface, and therefore reduced heat diffusion, gives rise to a higher temperature near the free surface, as demonstrated in Fig. 7b.

To further quantify the effect of increased temperature at the free surface, we have also examined an analytical model that assumes an absorbing spherical inclusion of radius *R* embedded in an infinite matrix of the same thermal properties. This analytical model is used to calculate the temperature at a distance *r*, where *r* corresponds to the free surface of the MLD at the first interface, as depicted in Fig. 7a. The absorption is assumed to be instantaneous, which is justified by the fact that *t*_width_ is much shorter than the relevant thermal diffusion time. We can therefore express the power absorbed per unit volume as

4 $$g(t)=(\sqrt{\pi /2}){g}_{\max }{t}_{{\rm{width}}}\delta (t-{t}_{{\rm{peak}}}).$$

The resulting temperature *T* at distance *r* is then given by (using an approach similar to that in ref.^30^)

5a $$\begin{array}{rcl}T(r > R,t) & = & -{\rm{TCF}}\frac{{g}_{\max }\frac{{\tau }_{p}}{2}\sqrt{\pi }}{\rho {c}_{p}}\frac{R}{r}{h}_{2}(\frac{t}{{\tau }_{{\rm{th}}}}),\\ {h}_{2}(u) & = & \frac{1}{2}(\sqrt{\frac{4u}{\pi }}\{\exp [-\frac{{(r/R-1)}^{2}}{4u}]-\exp [-\frac{{(r/R+1)}^{2}}{4u}]\}\\  &  & +\frac{r}{R}[{\rm{erfc}}\sqrt{\frac{{(r/R+1)}^{2}}{4u}}-{\rm{erfc}}\sqrt{\frac{{(r/R-1)}^{2}}{4u}}]),\end{array}$$

where the thermal diffusion time is

5b $${\tau }_{{\rm{th}}}={R}^{2}/D.$$

To quantify this temperature difference between the (more-precise) numerical predictions of the surface temperature to that of the analytical model (which assumes an infinite uniform matrix), we introduce in Eq. (5a) the “thermal concentration factor (TCF),” which represents the ratio between the temperatures at the surface (or other point) as predicted by the numerical and analytical models. Extensive comparisons between the numerical transient model and the analytical result of Eq. (4) lead to TCF = 2 for *r* at the free MLD surface and TCF = 1 for *r* at the first silica/hafnia interface.

The result in Eq. (4) may be used to assess the temperature at the free MLD surface or the first hafnia/silica interface when ${t}_{{\rm{width}}}\ll {\tau }_{{\rm{th}}}.$ This is easily verified in the range of *t*_width_, where the mechanism for type-II damage sites is observed (2 ps < *t*_width_ < 50 ps) since *τ*_th_ ~2.3 ns for *R*_defect_ = 40 nm. It must be noted that phase transitions and the temperature-dependent thermomechanical parameters were not considered in the models described above. The model should still provide, however, a good qualitative description of the trends and behaviors of the processes involved in the mechanism of type-II damage site formation. In particular, the energy deposition in the defect site can support, within a delay time of the order of 10 ns (depending on the depth of energy deposition), temperatures that approach or exceed the softening point of silica on the surface above the defect. This enables the pressurized hot material to be released via a venting pit that is located on the surface directly above the location of the defect. After ejection of the hot (in part, superheated) material, this venting pit has the appearance of a type-II defect.

### Modeling mechanism of type-III damage sites

Type-III damage sites consist of a conical crater with a high aspect ratio because of its very low depth of the order of 150 nm or less. Their shape is reminiscent of damage sites formed by gold nanoparticles (with diameter of the order of 20 nm) embedded under thin films having thicknesses up to 250 nm (ref.^31^). A two-stage material-removal mechanism was suggested^31^ involving initial material melting within the narrow region containing the gold nanoparticle and, upon temperature and pressure buildup, film fracture. In the current work, we hypothesize that the conical shape of the crater can be explained considering that the plasma formed generates high pressure originating from a small volume; this generates a plastically deformed zone analogous to that resulting from a sharp indentation. Specifically, sharp-point indentation in brittle substrates leads to the formation of radial cracks^32^ that can reach the surface under sufficient load, resulting in features very similar to those observed in type-III damage sites. This has been discussed in detail in the context of subsurface damage in fused silica resulting from polishing^33^.

**Figure 8:**
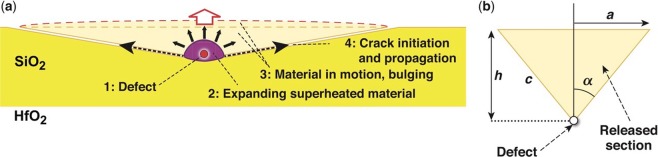
Depiction of the mechanism for formation of type-III damage sites. (**a**) An absorbing defect located near the surface gives rise to plasma formation during the laser pulse, resulting in the release of the overlying section via creation of a new surface. (**b**) Geometrical parameters considered when modeling type-III damage sites.

To consider modeling the underlying mechanism, we need to account for the fact that the energy absorbed by an incipient defect must be sufficient to create a conical crater. We therefore assume defect structures, absorbing laser energy according to their cross-sectional area, give rise to superheating of a small volume and a very localized high pressure (see Fig. 8a). Such defects must be located close to the surface in order to support the formation of the crater. The relevant geometrical parameters are depicted in Fig. 8b, where the defect is located at a depth *h*, i.e., a cone of inclined surface area with *α* being the cone apex half-angle. The area of the cone *A*_cone_ representing the newly generated surface is

6a $${A}_{{\rm{cone}}}=\pi \alpha {({h}^{2}+{a}^{2})}^{1/2}=\pi {h}^{2}/{\rm{\Omega }},$$

where the geometrical factor Ω is

6b $${\rm{\Omega }}={\cos }^{2}\alpha /\sin \,\alpha .$$

The necessary surface energy is *G*_c_*A*_cone_, where *G*_c_ is the critical energy release rate for silica, which can be estimated from the fracture toughness *K*_c_ and the Young’s modulus *E*_Y_ by

7 $${G}_{{\rm{c}}}={K}_{{\rm{c}}}^{2}/{E}_{y}.$$

Note that the critical energy release rate *G*_c_ differs from surface energy because *G*_c_ includes surface energy as well as the energy caused by any irreversible processes in surface creation (such as plasticity, dislocations, etc.). If the energy released by the superheated defect is *E*, the resulting crater will reach the surface when

8 $$E > {G}_{{\rm{c}}}{A}_{{\rm{cone}}}.$$

The energy *E* can be estimated by assuming that the defect of radius *R* absorbs the incident flux at a fluence equal to the experimentally observed damage threshold fluence (*F*_th_) according to its cross section, so that *E* = *F*_th_ (*π*^2^*R*). This leads to a maximum depth *h*_m_ for the defect such that the absorbed energy is sufficient to support the formation of a new surface and create the damage site as given by

9 $${h}_{{\rm{m}}}=R{({F}_{{\rm{th}}}{\rm{\Omega }}/{G}_{{\rm{c}}})}^{1/2}.$$

The AFM images from Fig. 3 show that the angle *α* is shallow, close to 86.5°, leading to Ω = 0.004. Using the value 0.7 MPa m^1/2^ for the critical stress intensity *K*_c_ in fused silica^34–36^, and Young’s modulus *E*_Y_ = 70 GPa, the critical energy release rate is *G*_c_ ~7 J/m^2^. For order-of-magnitude estimates, we use a laser-damage threshold value of LDT = 7.5 J/cm^2^ (ref.^12^) relevant to the mechanism for type-II damage sites, so that

10 $${h}_{{\rm{m}}}=6.4R.$$

For a defect radius of 20 to 40 nm, *h*_m_ is 127 to 255 nm, i.e., comparable to the values measured experimentally, as depicted in Fig. 3. In this mechanism, we have not shown the intermediate steps, namely the conversion of the absorbed laser energy *E* first into a pressure *p* within the defect mediated by the formation of the plasma. In addition, plasma expansion during the laser pulse would facilitate a transient increase of the absorbed energy during the laser pulse. Other mechanisms can be envisioned that may lead to crater formation, such as the growth of a tensile crack emanating from the plasma volume; this may form because of tensile hoop stresses in the surrounding fused silica, which can be further enhanced by geometrical considerations of the expanding plasma and/or phase instabilities in the plasma/material interface. In general, this model utilizes simple energy-balance considerations with reasonable energy-coupling factors. In this manner, it is possible to substantiate that type-III damage sites are superficial and originate from mechanical failure of the overlying material resulting from the energy absorbed by defects located at a maximum depth of the order of 150 nm.

### Mixed-Morphology Damage Sites

**Figure 9:**
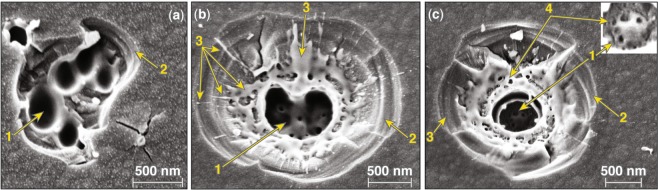
SEM images of damage sites formed by [(**a**,**b**)] 10-ps and (**c**) 50-ps laser pulses exhibiting mixed damage morphology: 1: damage pit; 2: mechanically cleaved crater sidewalls; 3: melted material and debris redeposits; and 4: porous material indicative of explosive boiling.

The experimental results and modeling of the three different types of damage sites presented in the previous sections describe observations near damage-threshold fluences for typical damage-initiation cases. However, damage can be initiated under more-complex conditions, resulting in localized energy density in the material that varies from the typical cases discussed above. Such complex damage-initiation conditions result in atypical, mixed-type damage morphologies. In this section, we discuss examples of such mixed-type morphologies that are infrequently observed (according to our experience) at near-damage-threshold conditions. Examples of such behaviors are shown in Fig. 9a,b.

Figure 9a shows the SEM image of a damage site formed at near-damage-threshold conditions under 10-ps irradiation. In this image, five defect (damage-initiation) sites are observed within an area of about 3 *μ*m^2^ as manifested by the remnant damage pits (example denoted as 1). The pressure generated in this region by the interacting damage events led to the removal of the overlying layer, creating side walls (2) similar to those observed in type-I damage sites. In particular, the appearance of the side walls indicates that the failure (rupture and creation of a new surface) was from mechanical stress, while there are also visual indications that melting was involved.

The damage site shown in Fig. 9b was also formed under 10-ps irradiation at the damage-threshold fluence. The image in this case suggests that there are two adjoining damage-initiation sites (1) that generated sufficient pressure to remove the overlying layer. The appearance of the side walls (2) suggests the formation via mechanical rupture. However, this damage site also contains substantial remnants of molten material (3) that include droplets of various sizes, ranging between 300 nm to less than 10 nm, that are deposited on the side walls (2) of the ruptured layer. The droplets commonly have a trailing conduit that indicates the radial trajectory away from the damage pit following ejection. The fact that the molten material overcoats the mechanically ruptured side walls shows that the molten material was deposited after the side wall was created. This in turn indicates that the pressure-induced mechanical failure (rupture) occurred earlier in the damage site-formation process, when the pressure is at its peak. Since heat diffusion is a slower process, the mechanical failure occurs while the overlying material is still unaffected by excessive heating.

The damage morphology shown in Fig. 9c was generated with 50-ps pulses at fluences ~10% above the damage threshold. This is the typical type-II morphology for pulses with durations between ~50 ps and 100 ps. Its morphology is very similar to that shown in Fig. 9b containing the damage pit (1), the ruptured side wall (2), and ejected molten material. However, the melted material located at the periphery of the damage pit as well as in the bottom of the pit (shown as inset with optimized image contrast) has numerous pores with diameters of the order of 50 nm or less, indicative of the presence of explosive boiling, similar to that described in detail elsewhere using nanosecond pulses^37^. The network of the porous structure is more pronounced and enlarged in mixed-type damage sites formed with 100-ps pulses (not shown). This type-II damage morphology for longer pulses arises from the greater energy deposited following plasma formation, which allows a strong energy coupling to form between the plasma and the laser pulse for about the second half of the pulse (assuming plasma formed near the peak intensity of the pulse).

### Transition from Volume Breakdown to Defect-Driven Damage Initiation

**Figure 10:**
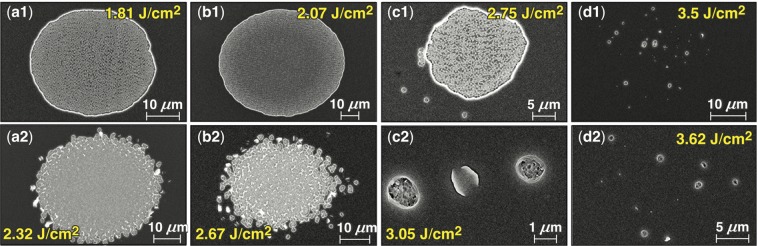
Paired SEM images obtained under (a1,a2) 0.6-ps excitation, (b1,b2) 1.3 ps, (c1,c2) 2.3 ps, and (d1,d2)] 2.9 ps under *p*- and *s*-polarized pulses, respectively. The corresponding laser fluences (J/cm^2^) are also shown.

Experiments were performed to determine the pulse length at which the transition from type-I damage morphology to type-II and -III morphologies occurs. The coating design used in this set of experiments (*s*-polarization mirror) exhibited damage initiation under subpicosecond irradiation (giving rise to type-I damage sites) in either the top (SiO_2_) layer under *s*-polarized pulses or the second (HfO_2_) layer under *p*-polarized pulses. Characteristic examples of SEM images of damage sites generated with a fluence just above the damage threshold under different pulse durations are shown in Fig. 10.

The images of damage sites shown in the upper row in Fig. 10 were obtained under *p*-polarized illumination, while the images in the lower row were obtained under *s*-polarization. This represents a set of four paired images of damage sites obtained under [(a1) and (a2) 0.6-ps], [(b1) and (b2) 1.3-ps], [(c1) and (c2) 2.3-ps], and [(d1) and (d2) 2.9-ps] excitation. These images reveal that damage initiation shifts from volume breakdown type I to defect-driven type II at ~2.3-ps for both polarization orientations of the pulse. Specifically, the SEM image of damage sites generated with 2.3 ps, *p*-polarized pulses shown Fig. 10c1 reveals the presence of both type-I damage sites (at the location of peak intensity of the laser pulse) as well as isolated (type-II) damage sites in the surrounding region. On the other hand, the SEM image of damage sites generated with 2.3-ps, *s*-polarized pulses (example shown in Fig. 10c2) reveal a mixed morphology where the isolated damage sites are observed but have a larger diameter than the typical type-II morphology. It must be noted that the edges of the type-I damage sites illuminated under *s*-polarized pulses are more irregular than those observed under *p*-polarized pulses. We postulate that this arises from the different depth of the crater in each case, which affected the shape of the blister formed. In the *s*-polarized case, the membrane is thinner and rupture boundaries can follow small variations in the electric field induced by either the laser beam or inhomogeneities in the coating. For pulses longer than 2.3 ps, type-II and type-III damage morphologies can be observed over the entire range of pulse lengths investigated and are often present on the same optic in close proximity.

**Figure 11:**
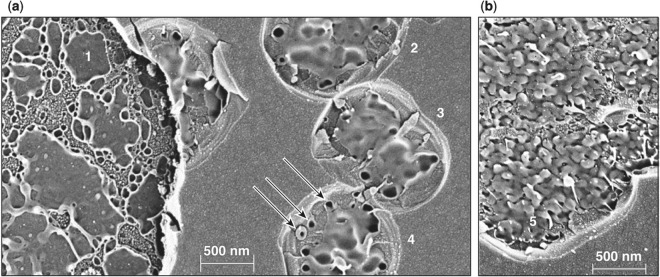
High-magnification SEM images of type I and type II damage sites under exposure to (**a**) *p*-polarized, 2.3-ps and (**b**) *s*-polarized, 600-fs pulses. Numbers 1–5 indicate different damage sites representing three different crater morphologies.

To better understand the crater morphology as a function of the depth of damage initiation and the transition from type-I to type-II damage sites, higher-magnification images of damage sites were analyzed. Figure 11(a) shows a region containing a section of a type-I damage site and a few type-II damage sites formed using 2.3-ps *p*-polarized pulses. This image is analogous to that shown in Fig. 10(c1). On the left side (denoted with numeric 1) is the type-I crater formed at the second hafnia/silica interface (the interface between first hafnia and second silica layers). The crater morphology is the same as that observed in Figs 1(b) and 10(a1, b1) and arises from the remnant re-solidified material after the cooling of the damage site. The corresponding type-I damage sites formed in the top silica layer under *s*-polarization have a different morphology as shown in the SEM image in Fig. 11(b). The crater morphology is the same as that observed in Fig. 10(a2, b2) and is representative of type-I damage formed in the top silica layer independent of laser pulse length (but <2.3 ps) and coating design. The crater has nanoscale projections similar, but smaller in size, to those observed in damage sites on the surface of fused silica optics under nanosecond laser irradiation that have been attributed to phase instabilities (see Fig. 51 in Manes *et al*.^38^). Furthermore, fibers observed at the tips of some of these projections indicate separation of liquid nanodroplets during explosive boiling, which is also a common characteristic in ns damage sites in fused silica.

The crater morphology is related to the material viscosity and other parameters (pressure, material flow dynamics, etc.) at the time of resolidification at the end of the material ejection process. Damage under *p*-polarized pulses initiates on the HfO_2_ side of the second interface, while only the top SiO_2_ layer is involved under *s*-polarized pulses. The melting and boiling temperature in SiO_2_ and HfO_2_ are very different leading to different material properties and dynamics of material ejection and subsequent cooling in each case. The type-II damage sites formed under irradiation by pulses having duration just above the transition (from type-I to type-II) pulse duration of about 2.3 ps have a different morphology. This is demonstrated in Fig. 11(a), in damage sites denoted with numbers 2, 3, and 4. AFM images indicate that the top ≈200-nm section of the first SiO_2_ layer has been removed and exposed a number of nano-pits located in close proximity (three characteristic examples are indicated with arrows). This crater morphology is very similar to those observed for pulses duration shorter than about 5 ps and very similar to those shown in Fig. 10(c2,d1,d2), and also 9a (defined as mixed morphology). There is evidence of melting and material flow through the nano-pit, which are characteristic traits of the type-II damage process. Although the origin (depth) of the nano-pits is not resolved, it is anticipated that they originate at the same depth as the bottom of the type-I crater (denoted with 1) in Fig. 11(a).

**Figure 12:**
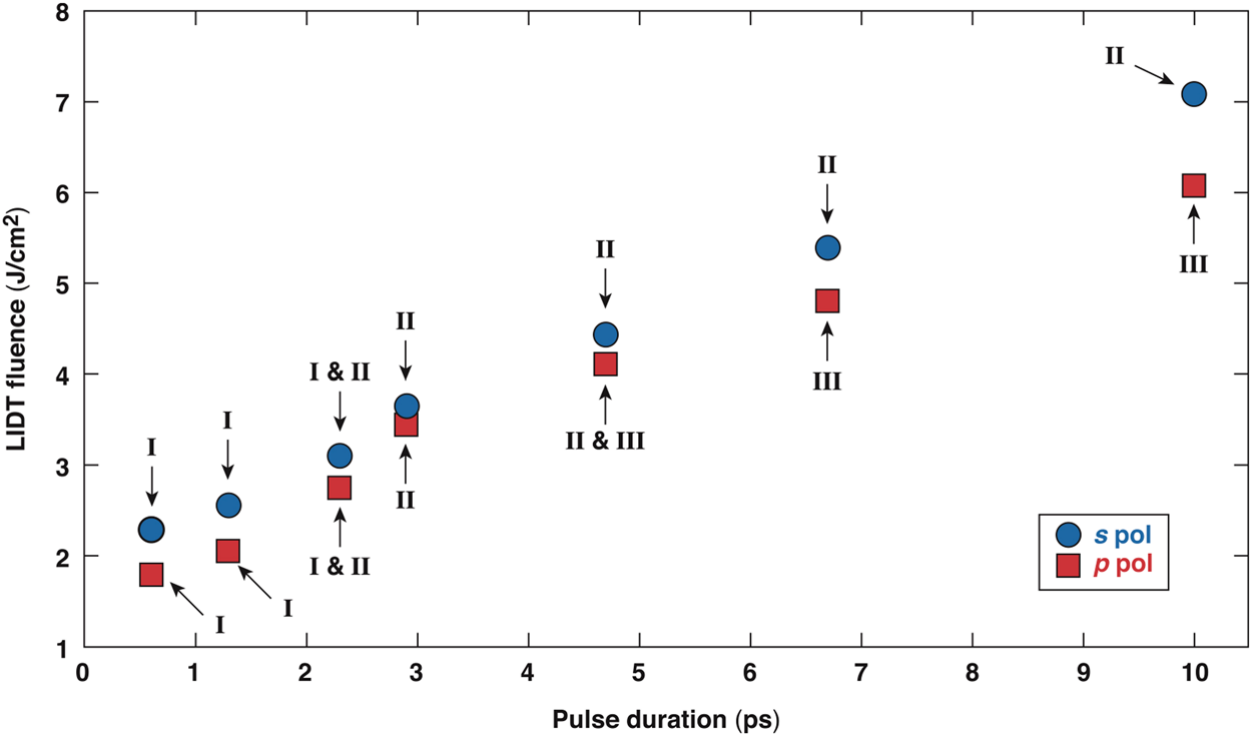
The damage threshold of sample 3 as a function of the pulse length under exposure to *s*-polarized (blue circles) and *p*-polarized (red squares) pulses. The arrow and numeric I, II, or III, correspond to the observation of type-I, type-II, or type-III morphologies, respectively.

As mentioned previously, the damage threshold increases with pulse length. Figure 12 shows the as-measured damage threshold as a function of the pulse duration for one of the samples studied (45°, *s*-polarized high reflector) under exposure to both *s*- and *p*-polarized pulses. The damage threshold results are accompanied by information on the type of damage morphology observed in each case at damage-threshold conditions indicated by the arrow and numeric I, II or III, corresponding to observation of type-I, type-II, or type-III morphologies, respectively. The results summarize the previous discussions regarding the morphology of the damage sites at different pulse durations. Specifically, type-I damage is observed for pulses shorter than 2.3 ps, while the presence of both type-I and type-II damage morphologies are observed at about 2.3 ps. For longer pulses, while type-II damage morphology is observed for *s*-polarized pulses, the behavior is more complex for *p*-polarized pulses; type-II and type-III damage sites are observed at damage-threshold conditions for an ~4.9-ps pulse, and only type-III morphologies are found for longer pulses. This behavior can be justified assuming that there are three damage-initiation mechanisms (yielding three different damage morphologies), with each mechanism having a specific initiation (LIDT) fluence threshold as a function of pulse length and polarization. For a specific pulse length and polarization state, each sample can theoretically be represented by three damage-threshold profiles: LIDT-I(*τ*), LIDT-II(*τ*), and LIDT-III(*τ*), where *τ* is the laser pulse length. For each pulse length, damage initiation is governed by the mechanism that presents the lowest LIDT. As a result, the damage threshold of the material is the lowest value between LIDT-I(*τ*), LIDT-II(*τ*), and LIDT-III(*τ*), and the damage-initiation mechanism (and associated damage morphology) can change as a function of pulse length.

## Conclusion

Damage initiation under short-pulse excitation is associated with (1) high-temperature–induced melting, material ejection, and structure modification and (2) pressure-induced mechanical damage. Depending on the type, size, and location of the damage-initiating defect as well as the laser-excitation parameters, one of these mechanisms dominates the morphology of the damage site at damage-threshold conditions. Defects present in both HfO_2_ and SiO_2_ layers can initiate damage; the excitation conditions (including the internal electric-field distribution) determine the “principal” damage-initiation mechanism.

Since damage is defined as an optically observable material modification, the damage-threshold irradiation conditions must support plasma formation and possess sufficient excess energy to cause observable material modification. This in turn suggests that nonobservable modifications may be present below the damage threshold, when the energy deposited is insufficient to produce a “damage site” involving an observable material modification. However, non-observable material modifications may still have a detrimental effect on the performance of the optic. This suggestion requires further investigation and may be more critical for optics exposed to femtosecond laser pulses, where the amount of energy deposited after plasma formation may be limited by the duration of the pulse.

This work suggests that there are three damage-initiation mechanisms in SiO_2_/HfO_2_ multilayer coatings under short-pulse laser excitation from 600 fs to 100 ps. Type-I damage is observed for pulses shorter than about 2.3 ps (under the excitation conditions used in this work), where crater formation is dominated by pressure-induced mechanical ejection of overlying material following plasma formation at the depth of peak electric-field intensity (EFI). Defect-driven damage initiation (type II and type III) is observed for pulse lengths from 2.5 ps to 100 ps. Type-II damage initiates in the first HfO_2_ layer at the depth defined by the local EFI peak, followed by a subsurface explosion involving melting and eventual venting of the evaporated material on a time scale of the order of 20 ns. Type-III damage is entirely confined to the top SiO_2_ layer at depths of less than ≈150 nm and shows no correlation with the local EFI peak. Type-III damage is associated with the release of material overlying a defect site caused by generated pressure that can originate only from a defect located at depths of less than about 200 nm.
